# Visualizing chemical functionality in plant cell walls

**DOI:** 10.1186/s13068-017-0953-3

**Published:** 2017-11-30

**Authors:** Yining Zeng, Michael E. Himmel, Shi-You Ding

**Affiliations:** 1 Biosciences Center, National Renewable Energy Laboratory, Golden, CO 80401 USA; 2 BioEnergy Science Center (BESC), Oak Ridge National Laboratory, PO Box 2008 MS6341, Oak Ridge, TN 37831 USA; 3 Department of Plant Biology, Michigan State University, East Lansing, MI 48824 USA

**Keywords:** Plant cell wall, Cell wall imaging, Biomass recalcitrance, Bioenergy, Lignocellulosic biomass, Stimulated Raman scattering, Atomic force microscopy, Fluorescence, Fluorescence lifetime imaging microscopy

## Abstract

Understanding plant cell wall cross-linking chemistry and polymeric architecture is key to the efficient utilization of biomass in all prospects from rational genetic modification to downstream chemical and biological conversion to produce fuels and value chemicals. In fact, the bulk properties of cell wall recalcitrance are collectively determined by its chemical features over a wide range of length scales from tissue, cellular to polymeric architectures. Microscopic visualization of cell walls from the nanometer to the micrometer scale offers an in situ approach to study their chemical functionality considering its spatial and chemical complexity, particularly the capabilities of characterizing biomass non-destructively and in real-time during conversion processes. Microscopic characterization has revealed heterogeneity in the distribution of chemical features, which would otherwise be hidden in bulk analysis. Key microscopic features include cell wall type, wall layering, and wall composition—especially cellulose and lignin distributions. Microscopic tools, such as atomic force microscopy, stimulated Raman scattering microscopy, and fluorescence microscopy, have been applied to investigations of cell wall structure and chemistry from the native wall to wall treated by thermal chemical pretreatment and enzymatic hydrolysis. While advancing our current understanding of plant cell wall recalcitrance and deconstruction, microscopic tools with improved spatial resolution will steadily enhance our fundamental understanding of cell wall function.

## Background

In our continuing endeavor toward producing renewable fuels and chemicals from plant biomass [1, 2], considerable effort has been devoted to genetically optimizing the amount, chemical composition, and basic structure of plant cell walls [3, 4]; as well as searching for better pretreatment and degradation methods [5, 6] to efficiently fragment biomass and produce fermentable sugars. No matter which direction is taken, these approaches break down the natural resistance of plant cell walls against deconstruction [1, 7, 8]. Cell wall chemistry and molecular architecture have already been proven to play a key role in the recalcitrance of energy plant cell walls [9]. At molecular level, the composition of cell wall layers, especially the distribution and migration of lignin during pretreatment, significantly impacts the local enzyme accessibility to cellulose [10–12]. A detailed understanding of the structural organization of cell wall chemistry at the microscopic and molecular scales is required in the search for effective biological and biochemical deconstruction of energy plant cell walls. By gaining critical insight into the fundamentals of wall structure, biomechanics, and reactions to stress and developmental modulations, microscopy helps us understand the manifestation of macroscopic observations. Novel imaging technologies provide unprecedented opportunities to probe the chemical functionality of wall polymers in the native state and during conversion to fermentable sugars. In this review, we provide a brief overview of recent progress by microscopy approaches toward understanding the chemical functionality of the plant cell wall, as well as its changes when subject to pretreatment and enzymatic degradation.

## Wall polymers of plant and their chemical functionalities

Energy plants, including grasses such as maize [13], sorghum (*Sorghum* spp.) [14], switchgrass (cultivars of *Panicum virgatum*) [15], miscanthus (*Miscanthus* and other *Miscanthus* spp.) [16], and energy cane (*Saccharum complex*) [17] and trees such as poplar (*Populus trichocarpa*, and other *Populus* spp.) [18], willow (*Salix* spp.) [19], pine (*Pinus* spp.) [20], and eucalyptus (*Eucalyptus* spp.) [21], are sustainable and renewable feedstocks for biofuels production. The cell wall comprises most of the plant’s dry weight and is composed primarily of three polymer components: cellulose, hemicellulose, and lignin. Dry plant in general contains 40 to 50% of cellulose, 15 to 25% hemicelluloses, 20 to 25% lignin, and 5 to 10% other components.

Polysaccharides are the principal components of plant cell walls and comprise their structural framework. Consisting of (1 → 4)-β-glucan units, cellulose is the most abundant plant cell wall polysaccharide [22]. Cellulose is synthesized by cellulose synthases at the plasma membrane by building β-glucan chains from UDP-glucose [23]. The cellulose microfibril grows from the non-reducing end by cellulose synthesis complex and is soon packed into an insoluble crystalline structure at the growing cell wall [24]. Cellulose is utilized to produce bioethanol and other chemicals by liberating glucose through chemical and biologically breakdown achieved by cellulolytic enzymes [25, 26] and fermentative microorganisms [27, 28]. Enzymatic hydrolysis of insoluble cellulose usually requires endoglucanases, exoglucanases (cellobiohydrolases), and β-glucosidases working in synergy [29, 30]. The heterogeneity [31] and insolubility [32] of the cellulose microfibril can produce a challenge for cellulolytic enzymes.

Hemicelluloses are the second most abundant heterogeneous polymers containing various monosaccharide subunits to form xylans, xyloglucan, mannans and glucomannans, and others [3, 33]. In plants, hemicelluloses are synthesized in the Golgi membranes [34]. It is also known that through covalent and non-covalent interactions with cellulose and lignin, hemicellulose contributes to strengthening the cell wall [35]. Unlike cellulose and lignin, hemicelluloses can be readily solubilized when treated by different temperatures and concentrations of alkali, acid, and other chemicals. Dilute sulfuric acid hydrolysis, for example, has proven to be a favorable process for solubilizing hemicelluloses and converting them into sugars [36, 37]. Besides chemical hydrolysis, enzymes (i.e., hemicellulases) are also used for hydrolyzing hemicelluloses [38].

Lignin and hemicelluloses are polymers matrixed around cellulose microfibrils and they are believed to be the main contributors to biomass recalcitrance [39, 40]. Lignin accounts for 20 to 35% of the dry weight of cell walls. In living plants, lignin is essential for the cell wall structural integrity by imparting stiffness and strength to the stem and root of the plant [41]. Lignin also contributes to the water proofing of conductive elements within the xylem tissue, which facilitates transport of water and solutes through the vascular system [42]. This waterproofing function helps protect plants against the pathogens, as well as the overall “biochemical invasion.” Lignin is a heteropolymer that normally contains three types of monomer units, syringyl (S), guaiacyl (G), and p-hydroxyphenyl (H) [43]. Recently, a new type of lignin polymer, caffeyl alcohol (C) lignin, has been proposed to be a potential candidate for renewable carbon fiber production [44]. Lignification is the final stages of cell differentiation in lignifying tissues. During lignification, lignin is deposited through free radical reactions within the carbohydrate matrix of the cell wall, infilling the inter-lamellar space by forming covalent bonds to the surrounding non-cellulosic carbohydrates [45]. As a result, lignin polymers present a chemically and structurally complex macromolecule that occurs predominantly in the xylem, tracheids, vessels, and fiber cell walls of land plants.

Cellulose, hemicellulose, and lignin entangle to form a complex matrix. One challenge for efficient utilization of cellulose, hemicellulose, and even lignin is to separate and depolymerize certain polymers without inadvertently impacting the others. Biomass recalcitrance is a collective phenomenon arising from both chemical and structural aspects of plants and cell wall across a wide range of length scales. At the molecular level, the extent of cellulose crystallinity [46] and the crosslinks between cellulose [47, 48], hemicellulose [49, 50], and lignin [51, 52] limit the penetration of enzymes/microbes to cellulose. At the structure level, the amount and location of lignin, the cell wall thickness, wall lamina, chemical composition, and porosity contribute heavily to recalcitrance. These factors vary by type of biomass and type of pretreatment. Therefore, there is a high demand for microscopic imaging tools.

## Imaging techniques for visualizing wall features

Numerous imaging techniques have been employed to investigate the content, concentration, and distribution of the biopolymer components within the plant cell wall. Traditional optical light microscopies, such as bright/dark field microscopy [53] and polarized light microscopy [54], and both transmission electron microscopy [55] and scanning electron microscopy [56] have been used to visualize plant cell wall morphologies. To probe with chemical specificity, the autofluorescence of the lignin polymer is traditionally adopted to image lignin distribution in the cell wall [57]. By using cytochemical staining and other labeling techniques, imaging the distribution of different carbohydrates is achieved [58, 59]. Even with limited chemical specificity, microscopic imaging of cell wall polymers has revealed heterogeneity in their distribution among different tissues, cells types, and locations on wall [60–62]. Non-deconstructive and non-invasive imaging techniques that are widely used in medical applications have also been applied for plant tissue imaging. Nuclear magnetic resonance imaging (MRI) has been used to image water distribution in plant tissue [63]. Positron emission tomography (PET) has been used to image ^11^C, ^13^N, ^15^O, and ^18^F isotopes in plant tissue [64]. X-ray computed tomography (CT) have also been applied to plant to produce 3D volumetric radiographic data [65]. Complementary to the above-mentioned in vivo imaging techniques (i.e., optical microscopy, MRI, PET, CT), mass spectrometry-based ex vivo imaging techniques (such as secondary-ion mass spectrometry, matrix-assisted laser desorption ionization) provide wide spectrum of chemical identity by harvesting appropriate samples from plant tissue [66]. For example, 3D time-of-flight secondary-ion mass spectrometry has been applied to image cellulose and lignin in plant cell wall [67]. More complex than bulk analysis, the rich information from microscopic imaging allows for more powerful analysis and quantitation to understand the chemical functionality of plant cell wall and its role in biomass conversion.

To precisely localize polymers in cell wall, recent endeavors have been devoted to improving chemical specificity and high spatial resolution. Non-destructive and label-free methods are capable of providing tissue/cell type-specific, compositional and structural information in air or under a fluid. Lignin’s autofluorescence can be used in fluorescence microscopy to image lignin directly. In addition to fluorescence emission intensity, fluorescence lifetime imaging microscopy (FLIM) also resolves lignin’s autofluorescence decay lifetime [68]. Compared to other fluorescence microscopies, such as scanning confocal microscopy or total internal reflection fluorescence (TIRF) microscopy, each pixel in a FLIM image contains the fluorescence decay rate information in addition to fluorescence emission intensity, thus providing an extra dimension of measurement [69, 70]. Besides fluorescence, lignin and non-fluorescent carbohydrates can also be imaged by chemical imaging microscopic techniques taking advantage of Raman vibrational fingerprints associated with their unique chemical structures [71, 72]. Chemical imaging of plant cell walls is now more efficiently performed by non-linear coherent Raman microscopies [73], such as coherent anti-Stokes Raman scattering (CARS) microscopy [74, 75] and stimulated Raman scattering (SRS) microscopy [74, 76]. The coherent Raman signal generated by these non-linear processes is so much higher than traditional confocal Raman that a 2048 × 2048 pixel resolution image can be obtained in less a few min [77]. Both of these non-linear coherent Raman microscopies have provided chemical mapping of cellulose [10, 77], lignin [74, 75, 77], and xylan [78] based on their unique vibrational frequencies. Considering that the spatial resolution of traditional optical microscopy is restricted by diffraction (best< 300 nm) [69], atomic force microscopy (AFM) is an ideal tool to study the topographic and physical property of cell walls at nanometer scale and in its native state with minimum sample preparation (no fixation, freezing, dehydration, or metal coating) [79].

## Plant cell wall architecture

The physicochemical properties of plant cell walls are determined not only by the chemical and physical properties of the individual cell wall polymers, but also by the spatial organization and interactions among them [80, 81]. Cell wall architecture plays a key role in determining recalcitrance. The plant cell wall has a multi-composite structure, consisting of several layers formed at different stages during cell growth and differentiation. The primary wall (PW), largely composed of cellulose, pectin, and hemicellulose, is formed first during plant cell growth stage [82]. While differentiating during growth, cells are expanded and elongated. Once the cell reaches its final size, the thickened secondary wall (SW) layers, accounting for most of biomass, are formed by deposit of wall substances onto the inside of the PW [83]. The parenchyma-type SWs (pSW) are thickened walls in parenchyma and collenchyma, which are normally in living cells; the sclerenchyma-type SWs (sSW) are secondarily thickened walls in highly differentiated cells, such as tracheary elements and fibers, which are elongated and dead cells [84–86]. Cell wall chemical composition varies dramatically in different cell types, different tissues, and different plant species. PW are non-lignified and they exist in some cells. Thickened SW are usually lignified and consist of multilayered structures from outside to inside: highly lignified compound middle lamellae (CML) containing middle lamellae and the primary wall, a thin S1 layer, a thick less-lignified middle layer S2, a thin inner layer S3, and a warty layer formed by lignin precursors. These lignified SWs account for the majority of the mass of plant biomass. The last stage of wall thickening also produces, inside the S3 layer, a warty layer which is resistant to a wide range of reagents [87].

**Fig. 1:**
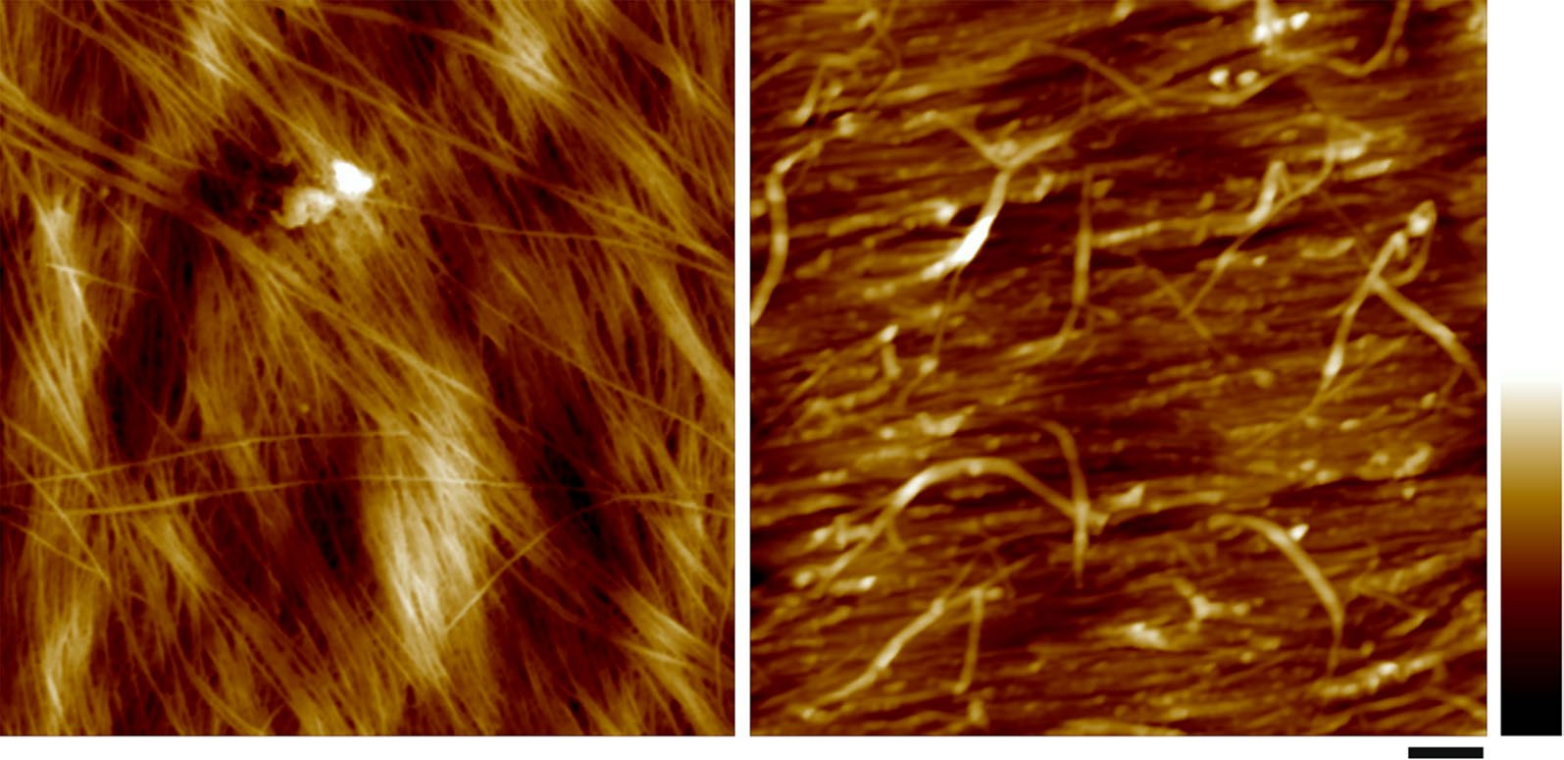
Atomic force micrograph of primary and secondary cell wall structure from maize. Left, cellulose microfibrils form varying sizes of bundles in PW. Right, cellulose microfibrils are heavily coated by matrix polymers in the S2 layer of SW. The image was taken from the cutting face of a vascular fiber cell from maize (reprinted from [10] with permission). Scale bar = 100 nm, color bar = 30 nm

Cellulose microfibrils form the scaffold of cell walls. The S1 layer is usually 300 to 400 nm thick and is composed of several lamellae of altered cellulose microfibrils with an orientation along the long axis of the cell [88, 89]. The S2 contains most of the cell wall cellulose and has a high content of parallel cellulose microfibrils [81]. AFM provides many useful details about cell wall cellulose microfibril organization at or near-physiological conditions [90–92]. At molecular level, cellulose forms rigid microfibrils which interact directly or indirectly with amorphous matrix polymers to form the composite cell wall lamellae. The cellulose microfibrils are often observed in AFM as bundles in the PWs that are composed of a number of cellulose elementary fibrils (CEFs) (Fig. 1). Although the size and cross-sectional shape of the CEF has not been determined, there is a general agreement about cellulose biosynthesis in vivo—where at least three cellulose synthase (CESA) isoforms are required to assemble a cellulose synthase complex (CSC) in the plasma membrane and together functions to synthesize the CEF [93, 94]. An 18-mer CESA complex has been proposed recently based on electron microscopy (EM) and freeze fracture techniques and computer simulation [95]. Assuming that all CESAs in the CSC are active and each synthesizes one glucan chain, it would result in an 18-chain microfibril. Other CEF models containing 36, 24 chains with hexagonal, square, or irregular cross-sectional shapes have also been proposed [96–101], further investigation in high spatial resolution imaging, particularly AFM is required to directly visualize the native structure of cellulose.

**Fig. 2:**
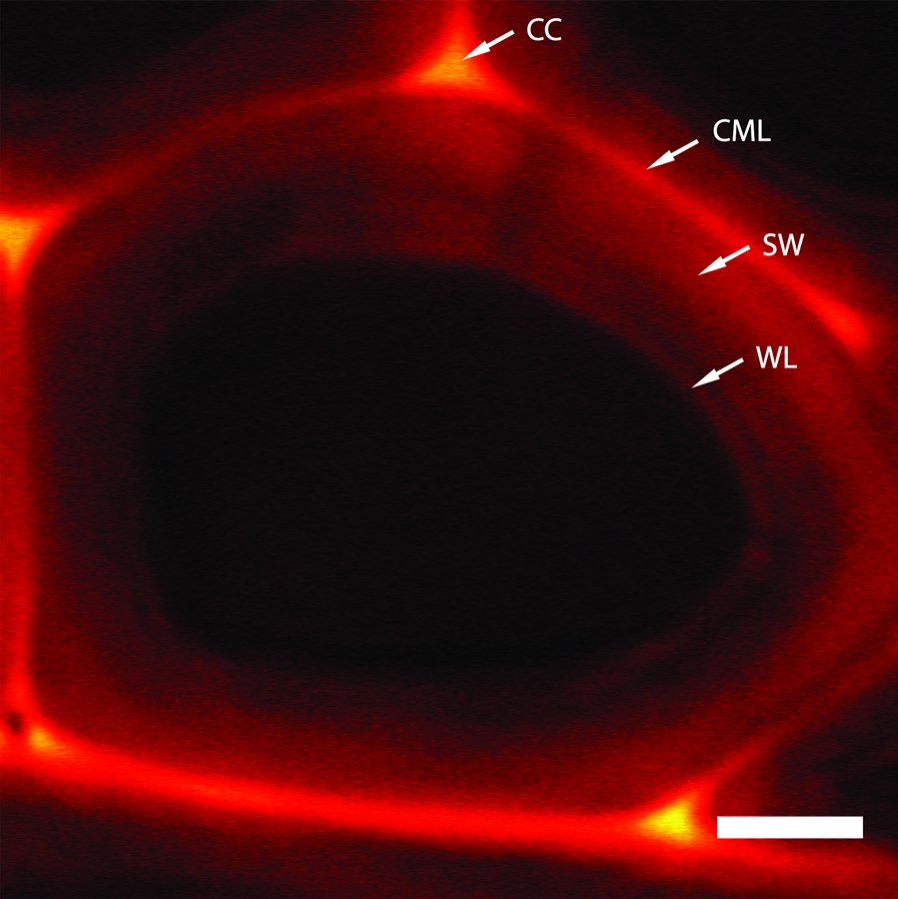
Lignin distribution in poplar tracheid cell wall imaged by stimulated Raman scattering microscopy by lignin’s aryl ring stretch at 1600 cm^−1^ (reprinted from [11] with permission). Lignin is unevenly distributed in cell wall layers. Highest lignin content is shown in the cell corner (CC), compound middle lamella (CML), and the warty layer (WL). Secondary wall (SW) has a lignin distribution gradient from outside (high) to inner side (low). Scale bar = 5 μm

Lignin has been conveniently imaged label-free by using stimulated Raman microscopy taking advantage of lignin’s strong Raman band at 1600 cm^−1^ [10, 11, 74, 75]. As shown in Fig. 2, the various lignin concentrations *in muro* are the result of unique stage of lignin synthesis during plant development. Lignification is the last stage of cell division, expansion, and elongation before cell death. In plants, lignin is synthesized through a radical polymerization process involving oxidative coupling of 4-hydrophenylpropanoids. This process can be either biologically programmed or triggered by environmental factors, such as stress conditions. Lignin monomers are produced inside cell membrane and then delivered to cell wall via mechanisms that are not completely understood. Lignification starts from the cell corner, accumulates in CML, and extends into PW, S1, S2, and S3, resulting in the lignin concentration gradient from high to low in these layers [11]. As shown in Fig. 2, the cell corner and CML have the highest lignin content. The adjacently lignified PW and S1 also have relatively high lignin concentrations. Moreover, the S2 and S3 are away from the lignification initialization sites and have less lignin content. The warty layer next to S3 is composed of highly cross-linked lignin precursors that are formed while the cell is in the final stage of lignification and death [11]. In biomass, the sSWs have fully lignified CML and warty layers; the pSWs are partially lignified and do not contain the S3 and the warty layer.

**Fig. 3:**
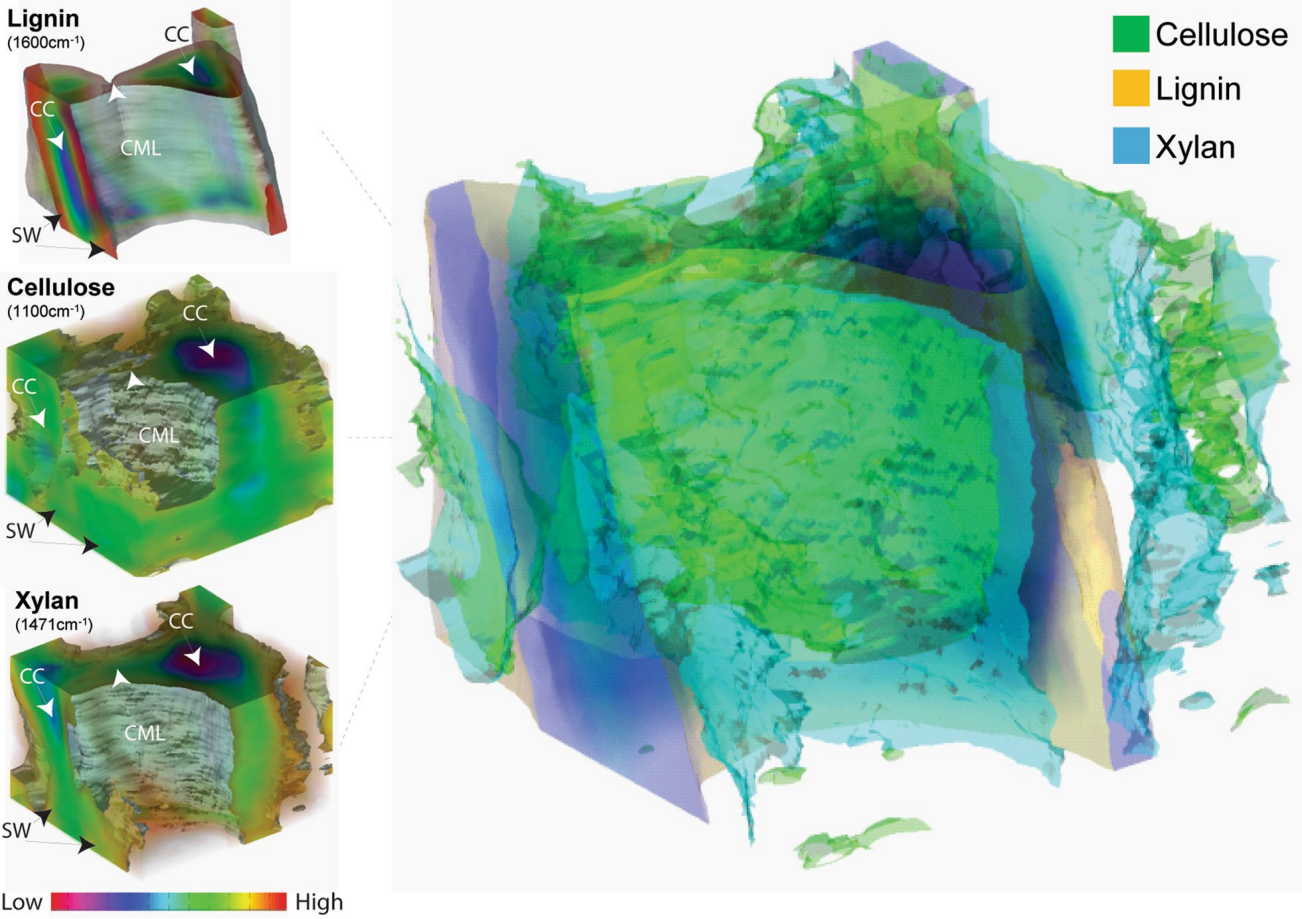
Lignin, cellulose, xylan in corn stover cell wall shown in individual 3D concentration distribution (left) and overlay of their isosurfaces (right) by reconstruction of stimulated Raman scattering microscopy sectioning scans. Lignin is more concentrated at cell corner and compound middle lamella, while cellulose and xylan are more abundant in secondary wall. Raman frequencies used for stimulated Raman scattering microscopy: lignin—1600 cm^−1^, cellulose—1100 cm^−1^, and xylan—1471 cm^−1^. *CC* cell corner; *CML* compound middle lamellae; *SW* secondary wall

Like lignin, cellulose has also been chemical imaged by its Raman band at 1100 cm^−1^ [71, 72]. However, chemical imaging of hemicellulose has been challenging. Due to the complex nature of plant materials, especially the chemical and structural similarities between hemicellulose (largely xylan) and cellulose, the utility of specific Raman vibrational modes that are unique to xylan has been debated. In a recent attempt to probe xylan-specific Raman bands, Zeng and coworkers [78] reported a novel approach based on combining spectroscopic analysis and chemical/enzymatic xylan removal. The authors identified several Raman peaks that are associated with xylan content in cell walls to be used for label-free in situ imaging of xylan. By using the above xylan signature Raman bands, along with those of lignin and cellulose, the 3D distribution of lignin, cellulose, and xylan (hemicellulose) in corn stover cell wall can be reconstructed through SRS section scanning (Fig. 3). Based on the 3D distributions, further material statistical analysis for their spatial distribution, such as volume, porosity, density, can be obtained.

## Wall change during pretreatment

Plant cell walls have evolved to resist natural breakdown from microbial, chemical, and mechanical challenges. Biomass recalcitrance is collectively determined by many factors, such as the content of cellulose/lignin/hemicellulose, the acetylation [102], methylation [103], hetero-polysaccharide deposition [104], inter-chain covalent bonding [105], H-bonding [106], van der Waals interaction [107], and finally pore size/density [108]. Note that to overcome recalcitrance, feedstocks in biochemical refinery will be routinely treated with acid and alkali at elevated temperature/pressure to expose useable polysaccharides to enzymes.

For decades, lignin has been viewed as the primarily contributor for biomass recalcitrance [11, 109]. In biorefinery, the amount and distribution of lignin throughout the cell wall determines the processing and eventual commercial utilization of energy plants. Since in the living plant lignin functions to provide cell wall with waterproofing, mechanical support, and resistance to breakdown, the chemical and structural characters of lignin are major barriers to deconstruction and utilization of lignocellulosic biomass. Therefore, one of the major strategies of biomass pretreatment has been aimed to remove lignin from the feedstock in order to enhance the accessibility of the polysaccharides to degradative cellulolytic enzymes and microbes. More recently however, a new view of lignin has emerged where it is not viewed purely as a barrier to polysaccharides utilization, but as a potentially useful and valuable component of biomass serving its own application for renewable chemicals [110]. Nevertheless, challenges of incorporating lignin conversion into the biorefinery scheme remain depolymerizing lignin and removing it from the cell wall without inadvertently generating any refractory form to processing.

As mentioned above, the SW constitutes most of the biomass dry weight and is the target for pretreatment. In the SW, lignin forms hydrophobic networks and is covalently bonded to hemicellulose. Layers of cellulose–hemicellulose and hemicellulose–lignin form a sandwich-like lamellae structure. In order to access these polysaccharides, specifically lignin in S2, must be removed. Lignin covalently binds to carbohydrates through benzyl ether bond [111], benzyl ester bond [112], phenyl glycoside bond [113], and acetal type bond [114] to form lignin–carbohydrate networks, connecting lignin firmly to the carbohydrate surrounding. Chemical cleavage of aromatic rings of lignin monomers, linkages between lignin units, ester or ether bonds between lignin and hemicellulose could all release lignin from polysaccharide network.

**Fig. 4:**
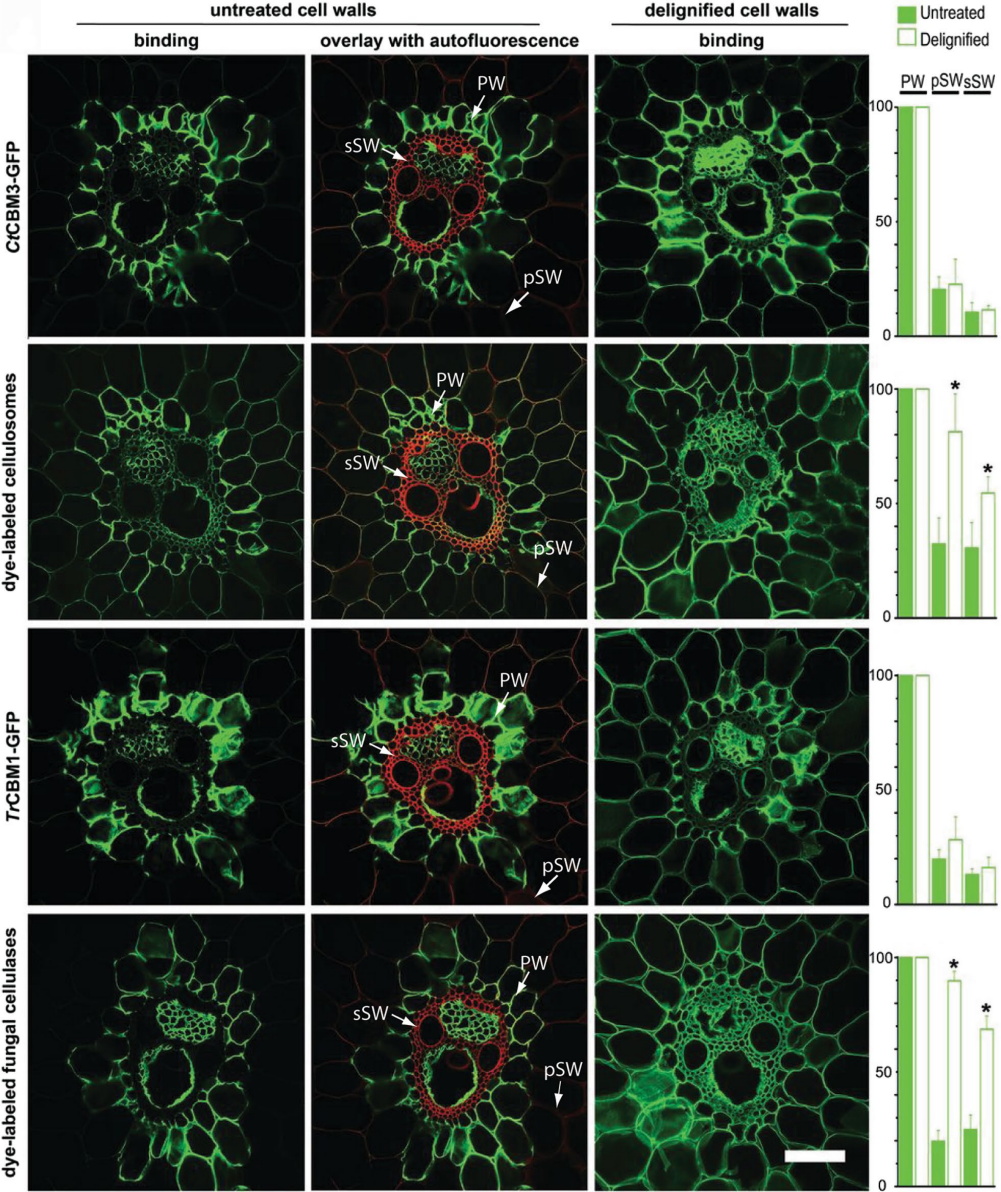
Confocal laser scanning microscopy of cell walls in transverse section of vascular bundle area when exposed to GFP-CBMs (reprinted from [10] with permission). CBMs specifically recognize cellulose, which is highly accessible in PWs, less accessible in pSWs, and non-accessible in sSWs. Lignin’s autofluorescence (red) and overlay images highlight the negative correlation between binding and lignin distribution. Delignification significantly increases cell wall accessibility to enzymes (paired *t* test, *P < 0.05). Histograms showing relative fluorescence intensity are expressed as percentages of fluorescence compared with the intensity of the labeled PW, which is designated as 100%. Delignified pSWs in the rind area were imaged in higher magnification. Scale bars = 50 μm

Pretreatments, such as dilute acid treatment at high temperature, can hydrolyze glycosyl bonds in hemicelluloses [115] so that lignin–carbohydrate complex (LCC) are formed and redeposited on the biomass surface as droplets, thus exposing cellulose. Some other pretreatment methods directly remove lignin. Pretreatments utilizing alkali, or other chemistries that directly hydrolyze the β-O-4 linkages in lignin, depolymerize the lignin polymer sufficiently so it can be efficiently extracted from the cell wall [116]. In order to remove lignin in the SW, the condensed lignin layers must be first fragmented, which may require the combined effects of mechanical, temperature, and chemistry, namely high severity treatment [11]. In plant cell wall, the inner face of the pSWs is non-lignified and already accessible, whereas in the sSWs, the S2 layer is sealed by CML and warty layer. By using GFP-tagged CBMs and enzymes, Ding and coworkers [10] visualized the accessibility of untreated cell walls. It was shown that the binding of CBMs and enzymes exhibits a strong negative correlation with lignin content in the cell wall layers. As shown in Fig. 4, CBMs and enzymes bind more to non-lignified PWs, less to pSWs, and negligibly to the condensed lignin in the “warty layer” in sSWs. Lignin removal enhanced overall binding of all CBMs and enzymes to lignified pSWs and sSWs. It was observed that accessibility of pSWs and sSWs to enzymes was enhanced more than that to CBMs, which could be explained by the increased accessibility of hemicelluloses to enzymes following lignin removal.

Pretreatment strategies may be adapted to different biomass feedstocks [117]. For example, dilute acid is sufficient for grassy feedstocks, because there are enough pSWs to allow acid penetration from the inner side of their walls. The same pretreatment condition may not work well for woody biomass, because wood chips are composed of predominately sSWs, which require much higher severity or different pretreatment methods that combine physical (milling or steam explosion) and chemical (delignification) processes.

It has now been widely accepted that pretreatment strategies need not specifically target lignin removal, but relocalize the lignin from its native context of close association with cellulose microfibrils [118]. One such example is thermochemical pretreatments that reach temperatures above the glass phase transition of lignin to cause effective physical and chemical perturbation to lignin network. As a result, the coalescence of lignin within cell walls and migration out of biomass during thermochemical pretreatments, accompanied by some subsequent re-deposition of lignin globules (lignin–carbohydrate complex, LCC) on cell wall surfaces has been observed [119]. Regardless of which pretreatment applied, one of their crucial attributes of all pretreatment strategies is the removal or relocalization of lignin to improve the accessibility of the carbohydrate in cell walls.

**Fig. 5:**
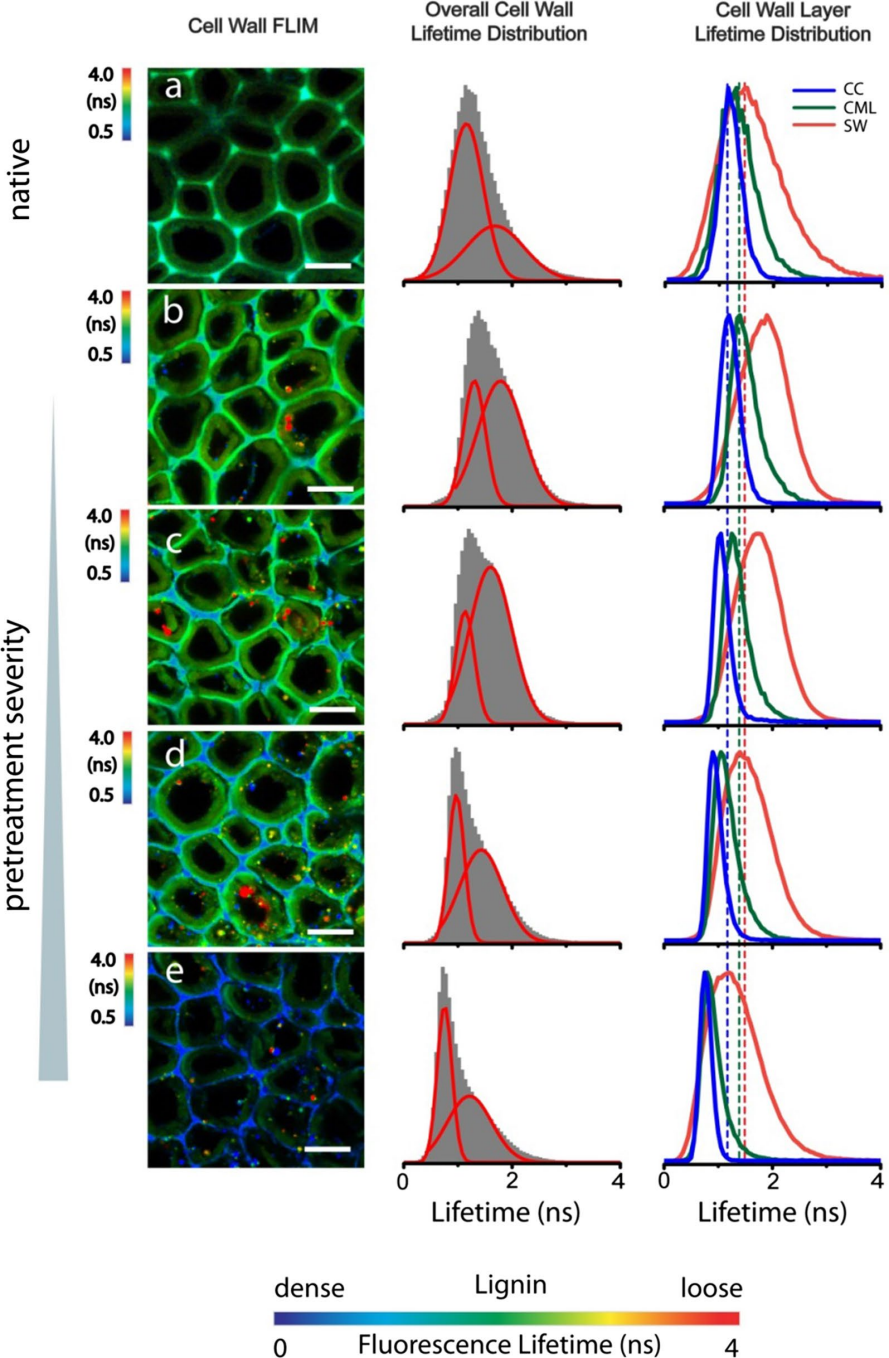
(Left Column) FLIM images of lignin in poplar cell walls from lignin’s autofluorescence (reprinted from [76] with permission). Comparison of untreated (**a**) and maleic acid-pretreated (**b**–**e**) poplar cell wall is shown. (Middle Column) Overall lignin fluorescence decay lifetime distribution across all the cell wall layers (gray). The two red curves are the two fitted *Gaussian* peaks by fitting the overall histogram, representing the dense and loose lignin in cell walls. (Right Column) Lignin fluorescence lifetime distributions within individual cell wall layer (*CC* cell corner; *CML* compound middle lamella; and *SW* secondary wall). Scale bar = 10 µm

Fluorescence lifetime imaging microscopy (FLIM) has been applied to track lignin’s fate in poplar during maleic acid pretreatment [76], a pretreatment previously showed lower sugar degradation than dilute acid pretreatment [120–122]. Zeng and coworkers found that the decay lifetime of lignin’s autofluorescence is correlated to the degree of condensation of lignin in the wall and the LCC produced by the maleic acid pretreatment. This lifetime is shorter for dense lignin and longer for loose lignin. In the FLIM images shown in Fig. 5, the dense lignin in the cell corner and compound middle lamella of poplar cell wall marked by shorter lifetime is clearly contrasted with the less dense lignin in the secondary wall shown in the longer lifetime case. Pretreatment produces LCC droplets containing various concentrations of lignin, as indicated by droplets displaying a wide range of fluorescence lifetimes in the FLIM images. Moreover, interesting evidence for lignin biosynthesis is obtained from FLIM images. In plants, I-lignification occurs during the early stage of secondary cell wall thickening at the cell corners, where a relatively high concentration of lignin monomers and peroxidases fill in an open space between cellulose microfibrils [123, 124]. Lignin is formed in the space and adhered between neighboring cells. I-lignification produces mostly dense lignin at the cell corner as confirmed by the short fluorescence lifetime observed at the cell corner. In compound middle lamella (containing no cellulose) and primary cell wall (containing mostly cellulose macrofibril), lignin appears to have slightly longer fluorescence lifetimes. In general, the cell corner and compound middle lamella contain dense lignin produced by I-lignification as evidenced by the relatively short fluorescence lifetime in FLIM images. Compared to I-lignification, S-lignification starts after the development of secondary cell wall. During S-lignification, lignin precursors permeate into the cellulose microfibrils framework in secondary cell wall producing relatively smaller amounts of lignin associated with large amounts of cell wall hemicellulose [124]. Lignin in SW produced by S-lignification is the less concentrated “loosely packed,” which is also identified by the longer fluorescence lifetime in FLIM images.

**Fig. 6:**
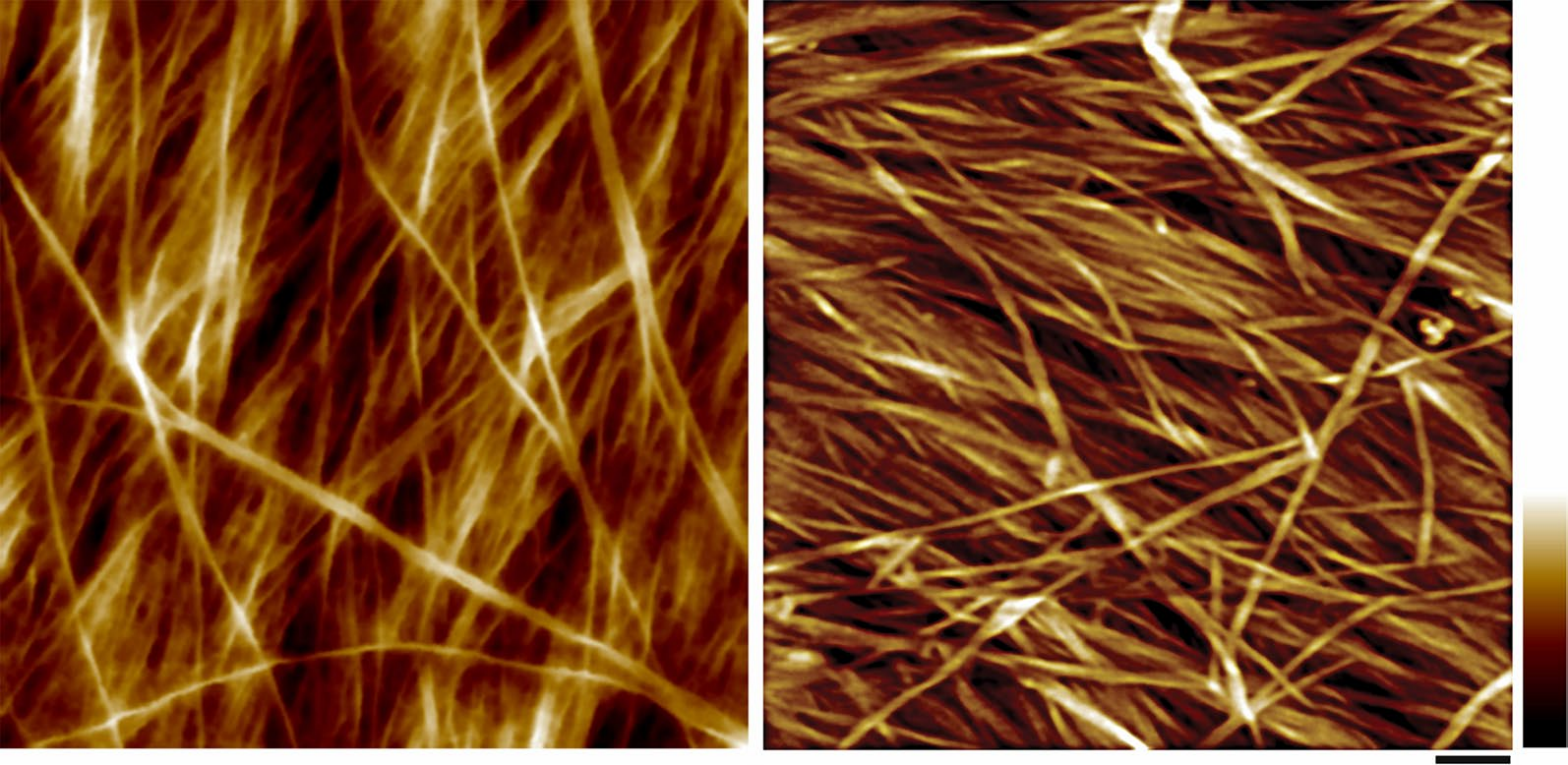
Atomic force micrograph of primary and secondary cell wall after delignification. Left, PW of maize parenchyma. Right, SW of maize vascular fiber cell. Delignification condition: 0.1 N HCl and 10% NaClO_2_ at 1% (w/v) biomass over night (reprinted from [10] with permission). Under this condition, lignin is nearly completely removed, and hemicelluloses are also partially removed. Scale bar = 50 nm. Color bar = 20 nm

Along with lignin removal, depending on biomass and pretreatment conditions, the depolymerization of hemicelluloses, physical separation of cell wall lamella, and creating porosity also contribute to enhance biomass accessibility. Enlarging the spaces among the cellulose microfibrils and creating pores are efficient to cellulase accessibility. AFM has been used to visualize enzymatic hydrolysis of isolated cellulose crystals [125, 126] and plant cell walls [10] in real-time (Fig. 6). It has been demonstrated that cellulases bind to and hydrolyze the hydrophobic faces of cellulose crystal [125, 126], which consequently result in “traffic-jam” in large crystals, while in the case of plant cell walls, the CEF is small, the enzyme accessibility to the substrate is the main rate-limiting factor that affects the efficiency of enzymatic hydrolysis [10]. In untreated biomass, the SWs are the major material of plant biomass, which is protected by lignin. Current cellulase mixture is not efficient in depolymerizing lignin, which physically impedes the accessibility of carbohydrate-active enzymes to access to the polysaccharides in the cell walls [10]. Therefore pretreatment is necessary to either remove lignin, such as dilute acid, or delocalized lignin, such as AFEX, so that cell wall polysaccharides, i.e., cellulose and hemicelluloses, can be hydrolyzed effectively by enzymes.

## Wall change during microbial/enzymatic conversion

Enzymatic digestibility of the cell walls is strongly negatively correlated to their lignin content [11, 109]. Even though non-lignified PWs are readily digested without pretreatment, the portion of non-lignified PWs in biomass is negligible. The non-lignified pSWs, such as pSWs collected in maize before reproductive growth, are also degradable, while the fully lignified sSWs in the same plant is not degradable [109]. When lignin in SW is selectively bleached (i.e., cellulose and hemicelluloses remain nearly unchanged), microscopic imaging of various types of cell walls during enzymatic digestion showed that all SWs are then found to be digestible at rates comparable to the PWs. Microscopic studies by Ding and coworkers found that in native cell walls, cellulosomes bind to the pSW innermost surface, the cell corners, and the plasmodesmata, whereas fungal cellulases penetrated into the pSW from the innermost surface. In these studies, non-specific binding of enzyme to native lignin was negligible [10].

The LCCs from pretreatment is also a factor affecting enzyme digestion. In the pretreated biomass, residual lignin normally forms LCC droplets or particles. Depending on pretreatment chemistry, lignin may or may not be chemically modified, and the composition of resulting LCCs may contain nearly pure lignin or significant amounts of polysaccharides—mainly hemicelluloses. Enzyme binding to the LCCs; therefore, relies on the relative content of polysaccharide and its morphological structure. For instance, in pretreatment in aqueous condition, such as dilute acid, the LCCs may form micelle-like structures where the lignin is the hydrophobic core and polysaccharides are surface displayed and thus attractive to non-productive binding of enzymes [127]. It has also been reported that lignin isolated from wood is more inhibitory to enzyme than that from herbaceous plant [128]; and lignin isolated from pretreated biomass, such as steam explosion, exhibited more inhibitory effect to enzymes than lignin isolated from non-pretreated raw biomass [129].

**Fig. 7:**
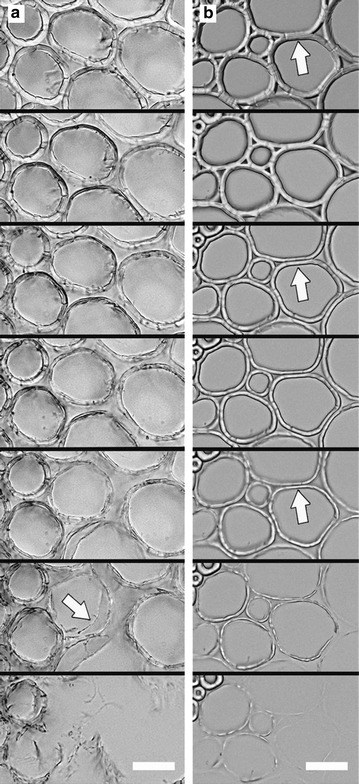
Delignified pSWs imaged in real-time during digestion at room temperature (reprinted from [10] with permission). Bright-field light microscopy of a transverse section digested (**a**) by cellulosomes for 7 days, showing wall fragmentation (white arrow), and (**b**) by fungal cellulases for 10 h, showing wall dissolving. White arrows in (**b**) indicate the wall’s innermost side. Scale bars = 50 μm

Although complete removal of lignin from biomass results in extremely digestible material as effective as corn starch, lignin removal must be performed at low temperature to avoid sugar degradation [130]. Microscopic studies showed that delignification of pretreated biomass with removal of most hemicellulose may result in significant reduction of enzyme digestibility [131], which could be attributed to the collapse and aggregation of the cellulose microfibril network [12], both of which reduce efficient enzyme penetration and rapid digestion. Corn stover delignified by acid chlorite at room temperature to retain cellulose and hemicellulose structure can be completely digested within 10 h at relative low loading of current commercially available cellulases [10]. Although ensemble solution measurement can only tell the difference in digestion rate, microscopic investigation discovered dramatically different mechanisms of cell wall digestion between cellulosomes and fungal cellulases [10]. As shown in Fig. 7, cellulosomes digested the cell wall in two steps: first separated the walls from CML and then dissolved the fragmented cell wall segments. By contrast, fungal cellulases digested in a more uniform rate across the whole cell wall.

**Fig. 8:**
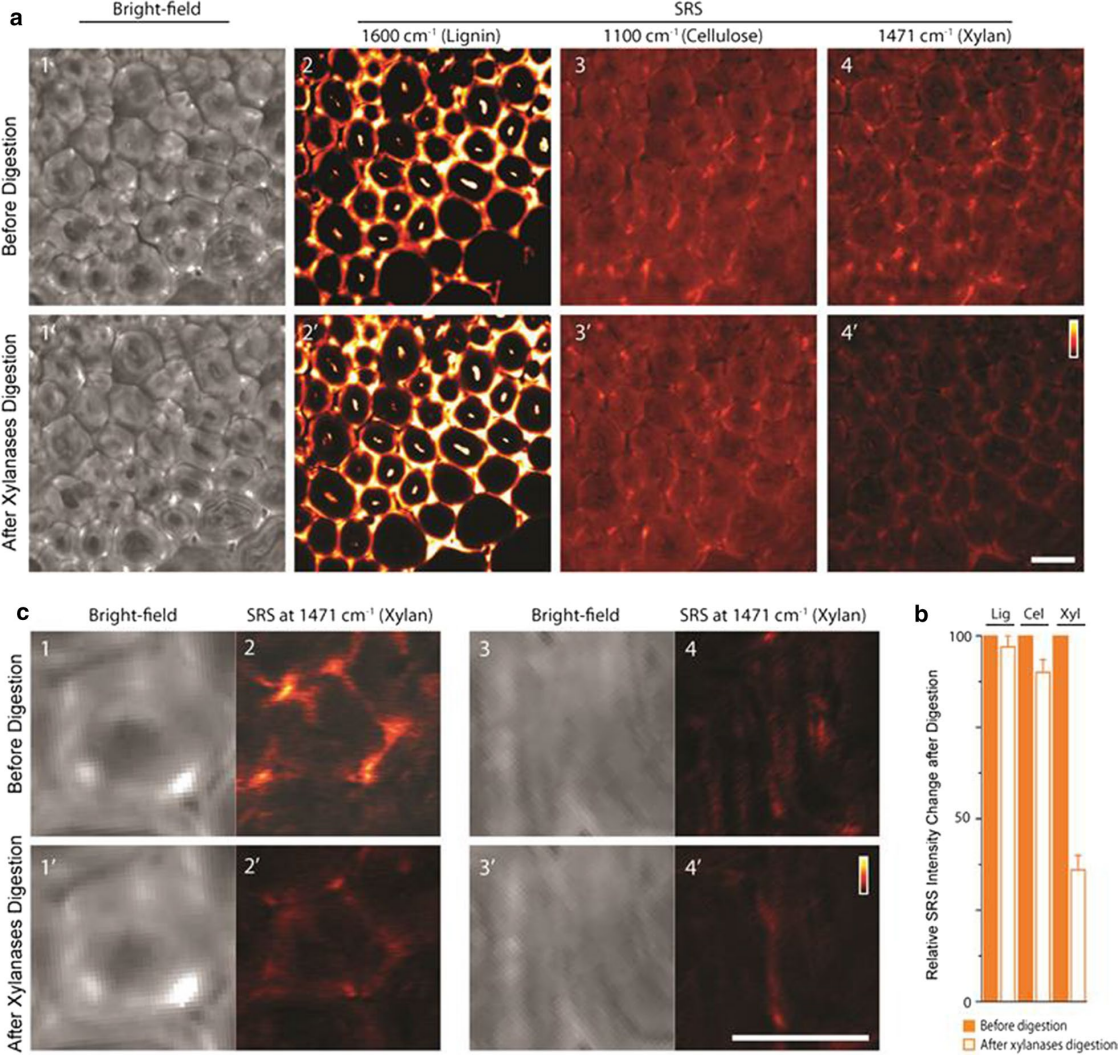
In situ imaging by stimulated Raman scattering microscopy of lignin, cellulose, and xylan in corn stover cell walls before and after xylan digestion. (Reprinted from [78] with permission). **a** Comparison of bright-field cell wall morphology and concentration of lignin, cellulose, and xylan following xylanase digestion. **b** Comparison of overall amount change measured from SRS images (before xylan digestion = 100%) (*Lig* lignin, *Cel* cellulose and *Xyl* xylan; error bars are from 5 repeat experiments). Lignin and cellulose are not affected, while xylan is significantly reduced due to xylanases digestion. **c** Zoom images of cell wall in two areas in vascular bundle region (*C1–C4* before xylan digestion; and *C1′–C4′* after xylan digestion) show significant xylan distribution changes in the cell wall due to the heterogeneous enzymatic digestion. Raman frequencies used for SRS imaging: lignin—1600 cm^−1^, cellulose—1100 cm^−1^, and xylan—1471 cm^−1^. Scale bar = 20 µm

Real-time imaging of lignin degradation by acidic chlorite pretreatment [77] and cellulose digestion by enzymes [10] has been achieved by stimulated Raman scattering microscopy. More recently, the impact on xylan distribution in cell walls by xylanase digestion was shown by both 2- and 3-dimensional display [78]. Zeng and coworkers used stimulated Raman scattering microscopy to image xylan, cellulose, and lignin following xylanase digestion (Fig. 8). Cell wall morphology and distribution of lignin, cellulose, and xylan in the same cell walls is compared before and after xylanase treatment. In contrast to lignin and cellulose Raman channels, dramatic concentration loss was observed for xylan. Importantly, besides significant reduction in concentration, xylan distribution in the cell walls was also altered by enzymatic digestion to take on “punctate type” morphology. Moreover, zoomed-in xylan images of two representative areas in the vascular bundle region showed significant xylan distribution changes due to the enzymatic digestion.

## Conclusions and future perspective

Correlative imaging through customized microscopies has been constructed to follow changes in the same plant tissue under near-physiological conditions or during actual pretreatment. High chemical and spatial resolutions have been achieved at tissue, cell wall, and molecular levels. We suggest that pretreatments should be developed to maximize lignin removal and maintain cellulose and hemicellulose intact. Energy plants with genetically modified lignins are especially promising because lignin extraction under mild conditions preserves polysaccharides, rendering them more readily digestible in the absence of lignin.

The major plant cell wall polymers and the interactions among them continue to be important topics in the design and utilization of energy plants. Interesting questions regarding plant cell wall polymers remain to be answered and imaging studies can contribute by addressing the following: How is lignin associated? How is hemicellulose assembled? Many aspects of microbial or enzymatic deconstruction of cell walls are also not well understood. For example, how do the large cellulosomal enzymes function to digest the diversity of substrate specificities found in cell walls? And what is the molecular organization of fungal cellulosomes?

The capability of label-free super-resolution imaging wall in three dimensions will be tremendously beneficial to unravel the organization of cell wall polymers. Super-resolution microscopic techniques have broken the traditional 200 to 300 nm Abbe limit for optical microscopy. Today, fluorescence-based super-resolution microscopies routinely achieve resolution at length scale ~ 10 nm. However, the spatial resolution in most Raman microscopies is still constrained by the optical diffraction limit. Surface-enhanced and tip-enhanced Raman spectroscopies can provide improved resolution, but it is difficult to extract quantitative information from the signal. Moreover, the plasmonic materials needed for signal enhancement may adversely affect the sample. By engineering the point-spread function, attempts have been made to improve the spatial resolution of CARS [132, 133]. Based on the photoswitching concept of stimulated emission depletion already applied in the fluorescence-based super-resolution imaging techniques, a stimulated Raman imaging technique known as “femtosecond stimulated Raman spectroscopy” has been reported to potentially achieve resolution < 50 nm [134].

## Abbreviations

MRI: nuclear magnetic resonance imaging
PET: positron emission tomography
CT: X-ray computed tomography
FLIM: fluorescence lifetime imaging microscopy
TIRF: total internal reflection fluorescence
AFM: atomic force microscopy
PW: primary wall
SW: secondary wall
pSW: parenchyma-type SWs
sSW: sclerenchyma-type SWs
CML: compound middle lamellae
CEFs: cellulose elementary fibrils
CESA: cellulose synthase
CSC: cellulose synthase complex
EM: electron microscopy
WL: warty layer
LCC: lignin–carbohydrate complex
CC: cell corner
CARS: coherent anti-Stokes Raman scattering
GFP: green fluorescent protein
CBM: carbohydrate-binding module
